# Factors Associated With Health-Related Quality of Life in Patients With Diabetic Foot Ulcer: A Cross-Sectional Study From Saudi Arabia

**DOI:** 10.7759/cureus.8658

**Published:** 2020-06-17

**Authors:** Mousab Al Ayed, Mutasem Ababneh, Asirvatham Alwin Robert, Nasser Al Misfer, Maria Cruz, Hesiel C Austria, Mohamed Al Dawish

**Affiliations:** 1 Department of Endocrinology and Diabetes, Prince Sultan Military Medical City, Riyadh, SAU; 2 Department of Physical Therapy, Prince Sultan Military Medical City, Riyadh, SAU

**Keywords:** quality of life, diabetic foot ulcer, general health, emotional well-being

## Abstract

Background and objective

Diabetic foot ulcers (DFU) have been shown to have a high impact on the patients’ perceived health-related quality of life (HRQOL). The aim of this study was to estimate the HRQOL and its related risk factors in patients with foot ulcers associated with type 2 diabetes mellitus (T2DM).

Methods

This cross-sectional study was performed on 81 patients with DFU, from January 2019 to July 2019 at the Prince Sultan Military Medical City (PSMMC), Riyadh, Saudi Arabia. The study population was purposively and conveniently chosen based on patients' availability during their regular and customary outpatient clinic visits. Using the Arabic version of the Short-Form 36-Item Survey (SF-36), these patients were interviewed and their HRQOL scores were was assessed. The SF-36 covered eight aspects of health such as physical functioning, body pain, limitations in the roles induced by physical health problems, limitations in the roles caused by personal or emotional problems, emotional well-being, social functioning, energy/fatigue, and general health perceptions.

Results

It was evident that age, gender, education, occupation, smoking, duration of diabetes, hypertension, dyslipidemia, body mass index (BMI), and the number of diabetes-associated complications, hypertension, and dyslipidemia significantly affected the patients' physical functions. The physical health of the patient was strongly influenced by gender, education, occupation, income, BMI, and the number of complications. The emotional health of the patient was affected by dyslipidemia, deformity, prior amputations, as well as BMI and glycosylated hemoglobin (HbA1c). The social standing of the patient was influenced by age, income, education, and occupation. The degree of pain experienced by the patient varied with age and the number of complications, as well as notable differences in their general health. The factors of age, education, occupation, income, and the number of diabetic complications induced several health changes in varying degrees. The patients with DFU revealed overall lower HRQOL relating to all the eight aspects of the SF-36.

Conclusion

The patients with DFU in Saudi Arabia generally revealed lower HRQOL. However, prospective and large-scale studies are required in the future to support these findings.

## Introduction

Diabetes mellitus (DM) is a public health challenge in the Arabian Gulf region, particularly in Saudi Arabia, which has been experiencing a disconcerting rise in the prevalence of DM in recent years. It is alarming to note that more than 25% of the adult population has been affected by this condition already, with projected numbers predicted to grow by two-fold or more by 2030 [1-3]. Over the past three decades, there has been an approximately 10-fold escalation of DM in Saudi Arabia. There is clear evidence to show that poorly managed diabetes and poor lifestyle lead to serious vascular complications [1-3].

Among the several complications that can affect a patient with diabetes, the most deleterious are those related to the foot. In fact, diabetic foot ulcer (DFU) is the single prevailing cause that induces the highest rates of morbidity in these patients, and the prevalence rates of DFU have been higher in Saudi Arabia compared to other countries in the Arab world [4-6]. Furthermore, patients with diabetic foot complications have a higher mortality rate when compared with diabetic patients without foot complications and the general population [5,7]. Evidence from several studies reveals that the risk of amputation is determined by the degree to which the patient is affected by DFU [8]. While one out of every six patients with diabetes experiences an ulcer during their lifetime in developed countries, patients in the developing nations, unfortunately, experience a much higher risk [8]. These diabetic foot lesions significantly affect the health and socioeconomic status of the patient, unfavorably influencing their quality of life (QOL), while inflicting heavy financial burden on their families [9,10].

The concept of health-related quality of life (HRQOL) covers the physical, psychological, and social aspects of patient health, which in turn are affected by his or her experiences, beliefs, expectations, and perceptions. All healthcare providers must necessarily be aware of the ways in which chronic diseases like DM impact the physical, emotional, and social aspects of the patient's life [11]. In patients with diabetes, particularly, multifactorial reasons induce lower scores of QOL. Such patients are usually of older age, likely to be overweight with a low likelihood of engaging in any routine physical exercise and are more likely to have illness-related complications as well as comorbidities like hypertension, coronary artery disease, and hypercholesterolemia. All these factors are linked to lower HRQOL scores [12,13].

Although Saudi Arabia recognizes DM as a major public health problem, data concerning HRQOL of diabetic foot patients is very limited due to a very high incidence of diabetic foot complications in the country. Therefore, this study aimed to estimate the HRQOL and its related risk factors in Saudi patients with foot ulcers associated with type 2 DM (T2DM).

## Materials and methods

This cross-sectional study was conducted among 81 (57 males, 24 females) patients with T2DM with DFU at Prince Sultan Military Medical City, Riyadh, Saudi Arabia, during January-July 2019. The study population was conveniently chosen, based on patients' availability in the course of their routine outpatient clinic visits. The inclusion criteria were as follows: patients between the ages of 18-70 years; patients with T2DM with the condition diagnosed for ≥1 year; and patients who were Saudi nationals. The exclusion criteria included patients having a history of psychopathology and medical instability, patients with visual, hearing, or cognitive damage, and those with type 1 diabetes and gestational diabetes.

Definitions of demographic variables and measurements

Patient demographics and DM history were noted. Based on their income, the patients were classified into the middle-income group [<10,000 Saudi Arabian Riyal (SAR) per month] or high-income group (≥10,000 SAR per month). Using a standardized sphygmomanometer, blood pressure measurements were recorded by a trained nurse, maintaining the patient in a sitting posture, with the arm kept at heart level and after five minutes of rest. Elevated systolic (≥140 mmHg) or diastolic (≥90 mmHg) blood pressure was diagnosed as hypertension.

In this study, detailed data were gathered on diabetes-linked complications like diabetic nephropathy, diabetic neuropathy, diabetic retinopathy, diabetic cardiomyopathy, coronary artery disease, peripheral vascular disease, and stroke. Nephrologists defined diabetic nephropathy as the condition where the value of microalbuminuria was ≥30-299 mg in the 24-hour urine collection sample or where macroalbuminuria was ≥300 mg in the 24-hours urine collection sample; the data were drawn from the patient records. As diabetic neuropathies are heterogeneous, several parts of the nervous system are affected and patients manifest a plethora of clinical symptoms, which could be either focal or diffuse. Retinopathy was identified based on the American Academy of Ophthalmology criteria, and the patients were classified into those having nonproliferative diabetic retinopathy and those with proliferative diabetic retinopathy.

The glycosylated hemoglobin (HbA1c) was analyzed by using the COBAS INTEGRA 400 plus/800 analyzers (Roche Diagnostics, Basel, Switzerland) at the Prince Sultan Military Medical City (PSMMC) central laboratory. The control of the lipid profile constituents employed in this study is based on the National Cholesterol Educational Program Adult Treatment Panel III guidelines and the recommendations of the American Diabetes Association. The definition of the family history of DM was limited to the occurrence of DM among patients' first-degree relatives.

SF-36

The HRQOL scores of the patients were estimated by interviewing them, employing the Arabic version of the Short-Form 36-item survey (SF-36). Privacy and confidentiality were maintained by ensuring that each patient was interviewed separately in a room. No pertinent mean differences were observed between the Arabic and English SF-36 questionnaires pertaining to Saudi culture [14]. The SF-36 covered eight aspects of health: physical functioning, body pain, limitations in the roles induced by physical health problems, limitations in the roles caused by personal or emotional problems, emotional well-being, social functioning, energy/fatigue, and general health perceptions. One separate item was added, which provided some evidence of perceived change in health. All 36 items were adapted from various instruments and were completed by patients involved in the Medical Outcomes Study, an observational study of the dissimilarities in the styles of physician practice and patient outcomes in various healthcare delivery systems [15]. For illiterate patients, the questionnaires were answered with two witnesses present.

The Likert method of summated ratings was the basis on which the SF-36 health survey items and scales were constructed. Every single answer was scored. The raw scale scores for each health concept were acquired by totaling the answer scores. They were then converted into a 0-100 scale, in which the higher scores indicated higher functioning, well-being, and state of health. The SF-36 was shown to be reliable and valid for both type 1 and type 2 DM patients [14].

Statistical analysis

Data were analyzed using Microsoft Excel (Microsoft Corp., Seattle, WA) and SPSS Statistics version 22 (IBM, Armonk NY). Apart from the descriptive analysis t-test, one-way analysis of variance (ANOVA) and Tukey post hoc tests were also performed to identify the differences and to facilitate comparisons to be made among the groups tested. The HRQOL-related variables were studied using linear regression analysis. A p-value of <0.05 was considered statistically significant.

## Results

Table 1 lists the demographic data of the participants (gender, age, marital status, education level, treatment type, income, and employment). The study population [57 (70.4%) males and 24 (29.6%) females] had a mean age of 53.1 ±11.4 years. The mean duration of the diagnosis of DM was 11.8 ±8.4 years. Most patients (44, 54.3%) had school-level education, received insulin treatment (42%), belonged to the low-income group (69.1%), had never smoked (66.7%), and had a family history (93.8%).

**Table 1:** Sociodemographic characteristics of the study population SAR: Saudi Riyal

Variable	Number	%
Gender		
Male	57	70.4
Female	24	29.6
Age		
30-40 years	22	27.2
41-50 years	16	19.8
51-60 years	29	35.8
61-70 years	14	17.3
Marital status		
Married	73	90.1
Unmarried	8	9.9
Education		
None	22	27.2
School	44	54.3
College	15	18.5
Treatment type		
Diet	6	7.4
Oral	11	13.6
Insulin	34	42.0
Oral + insulin	30	37.0
Occupation		
Employed	24	29.6
Unemployed	57	70.4
Income		
<10,000 SAR	56	69.1
≥10,000 SAR	25	30.9
Smoking		
Current	14	17.3
Never	54	66.7
Past	13	16.0
Duration of diabetes		
<10 years	14	17.3
≥10 years	67	82.7
Family history of diabetes		
Yes	76	93.8
No	5	6.2

Table 2 lists the clinical characteristics of the study population. While a significant portion of the patients fell within the uncontrolled diabetes group (74.1%), the others were categorized as hypertensive (72.8%), those with dyslipidemia (82.7%), obese (46.9%), having three or more complications (33.3%), having a deformity (54.3%), and those with prior amputation (29.6%).

**Table 2:** Clinical characteristics of the study population HbA1c: glycosylated hemoglobin; BMI: body mass index

Variable	Number	%
HbA1c		
<7%	21	25.9
≥7%	60	74.1
Hypertension		
Yes	59	72.8
No	22	27.2
Dyslipidemia		
Yes	67	82.7
No	14	17.3
BMI		
Normal	20	24.7
Overweight	23	28.4
Obese	38	46.9
Complications		
1 complication	25	30.9
2 complications	29	25.8
≥3 complications	27	33.3
Deformity		
Yes	44	54.3
No	37	45.7
Previous amputation		
Yes	24	29.6
No	57	70.4

**Figure 1:**
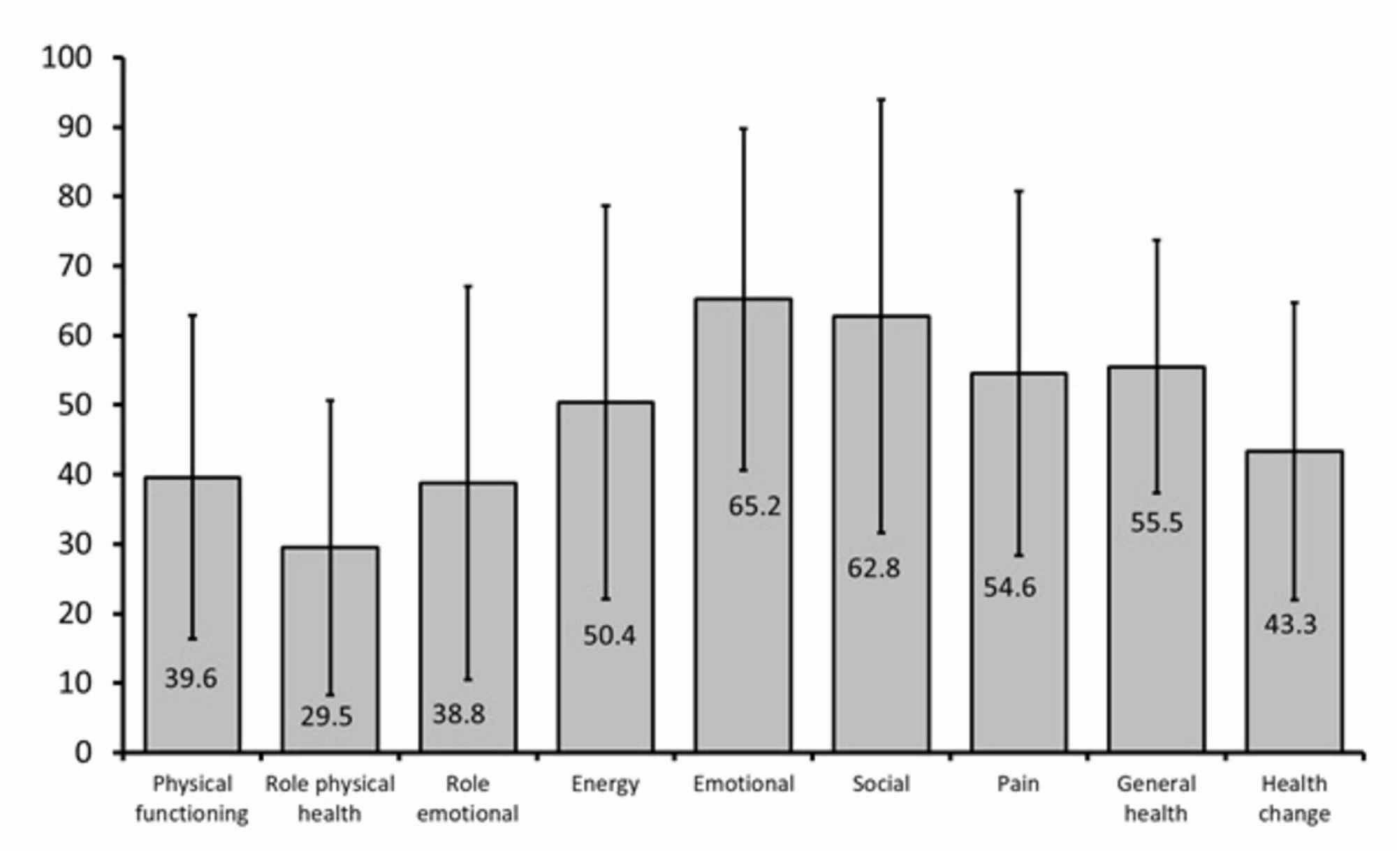
Health-related quality of life score of the study population* *Based on Short-Form 36-Item Survey

Figure 1 shows the total HRQOL scores of the study population for different parameters. The results of the patients with DFU revealed lower total HRQOL scores for all eight aspects in the SF-36 and the additional item (perceived change in health).

Tables 3, 4 show the manner in which the demographic and clinical factors affect the QOL. From the univariate analysis, it was seen that the independent factors such as age, gender, education, occupation, smoking, duration of diabetes, hypertension, dyslipidemia, body mass index (BMI), number of diabetes complications, hypertension, and dyslipidemia induce significant differences in physical function. The role of physical health is strongly influenced by gender, education, occupation, income, BMI, and the number of complications. The part played by emotions is influenced by the level of education, occupation, income, BMI, and HbA1c. Also, age, income, BMI, and the number of complications cause vital differences in emotional well-being. Social standing is impacted by education, income, dyslipidemia, deformity, and prior amputations. Significant variations in pain are found to be influenced by education, occupation, and income, while the general health is impacted by age and number of complications. Age, education, occupation, income, and the number of complications are found to precipitate health changes. In fact, the total QOL scores show significant differences according to age, education, occupation, income, and the number of complications.

**Table 3:** Influence of demographic variables on health-related quality of life of the study population Values are presented as mean ± standard deviation (analysis performed using t-test, one-way analysis of variance, and Tukey post hoc test) *Comparison of two groups: *gender: male vs female; *marital status: married vs unmarried; *occupation: employed vs unemployed; *income: <10,000 SAR vs ≥10,000 SAR; *duration of diabetes: <10 years vs ≥10 years; *family history of diabetes: yes vs no Comparison of more than two groups: age: #30-40 years vs 41-50 years, †30-40 years vs 51-60 years, ‡30-40 years vs 61-70 years, ƒ41-50 years vs 51-60 years, ¶41-50 years vs 61-70 years, ‡51-60 years vs 61-70 years; education: #none vs school, †none vs college, ‡school vs college; smoking: #current vs never, †current vs past, ‡never vs past SAR: Saudi Riyal

Variable	Category	Physical functioning	Role physical health	Role emotional	Energy	Emotional	Social	Pain	General health	Health change
Gender	Male	46.5 ±26.7	37.8 ±39.4	53.2 ±44.3	51.9 ±26.4	64.9 ±25.5	67.7 ±29.3	65.3 ±26.4	57.7 ±19.5	55.6 ±21.7
	Female	23.5 ±17.8*	11.1 ±5.2*	15.2 ±14.0	48.5 ±15.9	68.5 ±18.1	51.5 ±28.3	43.2 ±25.9	54.9 ±17.0	38.3 ±11.3
Age	30-40 years	51.1 ±25.4	29.6 ±9.5	51.5 ±26.8	49.7 ±28.0	59.2 ±25.0	63.6 ±29.3	63.9 ±25.0	49.5 ±19.4	52.3 ±20.1
	41-50 years	40.6 ±26.3	25.0 ±12.9	50.0 ±38.6	55.9 ±14.4	79.0 ±16.5#	71.0 ±24.8	61.5 ±22.1	62.1 ±17.8#	55.7 ±15.9
	51-60 years	40.8 ±32.7	31.9 ±14.7	35.6 ±22.6	53.7 ±23.7	66.7 ±23.5	62.5 ±29.5	56.0 ±33.9	62.5 ±18.6†	51.2 ±24.2
	61-70 years	18.5 ±17.2‡¶‡	14.5 ±9.2	31.1 ±22.1	41.0 ±24.0	60.2 ±23.9¶	53.5 ±36.1	53.2 ±25.8	50.6 ±14.4‡	40.3 ±17.1¶
Marital status	Married	40.7 ±29.1	29.5 ±12.2	41.6 ±35.0	51.5 ±23.8	65.4 ±23.6	61.1 ±30.0	59.7 ±28.5	56.6 ±18.8	50.8 ±21.4
	Unmarried	30.6 ±27.4	3.1 ±8.8*	45.8 ±26.9	45.0 ±23.6	71.5 ±23.9	79.6 ±23.0	50.0 ±22.2	58.7 ±19.9	48.0 ±13.5
Education	None	22.7 ±17.9	2.3 ±2.6	16.6 ±12.1	48.1 ±17.6	66.5 ±21.9	59.1 ±29.9	45.9 ±27.4	51.3 ±15.1	39.1 ±13.1
	School	47.8 ±27.9#	27.8 ±14.1#	45.4 ±36.0#	50.1 ±26.8	63.6 ±25.3	57.9 ±30.8	57.8 ±37.0	57.9 ±17.8	51.1 ±21.7#
	College	41.0 ±23.3†	60.2 ±44.7†‡	69.0 ±40.4†	57.3 ±22.1	72.2 ±20.4	83.3 ±16.1†‡	80.3 ±18.6†‡	62.0 ±24.8	65.7 ±17.5†‡
Treatment type	Diet	24.1 ±20.5	8.3 ±10.4	16.6 ±10.8	50.8 ±24.5	54.6 ±14.8	52.1 ±24.2	47.5 ±29.5	59.1 ±11.1	39.2 ±19.3
	Oral	44.5 ±36.6	22.8 ±16.0	45.4 ±37.7	58.6 ±13.9	74.1 ±20.7	63.7 ±29.3	65.2 ±28.7	57.2 ±22.4	53.9 ±18.3
	Insulin	44.2 ±28.8	28.0 ±11.1	37.3 ±24.6	52.2 ±25.7	70.1 ±23.3	67.6 ±29.7	60.1 ±27.5	54.7 ±21.3	51.8 ±21.2
	Oral + insulin	36.0 ±25.2	30.9 ±18.6	51.1 ±34.3	46.6 ±24.2	60.6 ±40.9	59.6 ±31.4	57.1 ±28.7	58.8 ±15.9	50.1 ±21.6
Occupation	Employed	54.1 ±23.4	42.9 ±33.9	68.1 ±39.6	56.8 ±21.3	66.6 ±19.0	72.3 ±23.3	70.0 ±23.5	55.6 ±20.2	60.8 ±13.9
	Unemployed	33.6 ±29.0*	20.2 ±3.7*	31.0 ±22.6*	48.4 ±24.4	65.7 ±25.3	58.9 ±31.4	54.0 ±28.5*	57.4 ±18.3	46.1 ±21.6*
Income	<10,000 SAR	38.3 ±30.9	18.3 ±13.1	30.9 ±22.0	50.5 ±24.6	62.3 ±25.2	56.6 ±31.8	54.2 ±30.0	54.4 ±17.8	45.7 ±20.9
	≥10,000 SAR	43.0 ±23.8	46.1 ±39.1*	66.7 ±41.7*	51.8 ±22.1	74.2 ±16.8*	77.0 ±18.2*	68.5 ±19.9*	62.4 ±20.1	61.2 ±16.0*
Smoking	Current	59.6 ±24.5	32.1 ±19.7	42.9 ±30.0	50.0 ±27.0	60.8 ±28.6	58.9 ±35.2	56.2 ±31.7	57.1 ±21.6	52.2 ±24.3
	Never	36.9 ±29.3#	23.1 ±14.6	39.5 ±25.8	52.2 ±21.8	66.9 ±21.7	63.9 ±28.1	58.1 ±25.8	56.4 ±18.9	49.6 ±18.6
	Past	30.4 ±23.5†	36.9 ±14.9	51.5 ±38.1	46.5 ±29.1	67.7 ±26.5	63.5 ±32.8	64.2 ±33.9	58.8 ±16.1	52.4 ±26.3
Duration of diabetes	<10 years	55.3 ±28.1	32.2 ±8.4	54.7 ±39.9	54.2 ±34.5	64.5 ±32.3	64.2 ±27.2	71.0 ±25.0	58.2 ±26.7	56.8 ±27.2
	≥10 years	36.4 ±28.2*	25.8 ±17.1	39.3 ±23.7	50.2 ±21.1	66.3 ±21.6	62.6 ±30.5	56.1 ±28.0	56.6 ±16.9	49.2 ±19.1
Family history of diabetes	Yes	39.3 ±29.1	25.4 ±16.8	41.2 ±35.7	51.3 ±23.8	65.8 ±24.0	63.3 ±30.0	59.4 ±28.1	57.4 ±18.8	50.4 ±21.1
	No	46.0 ±27.4	50.0 ±39.5	53.3 ±29.8	45.0 ±23.4	68.0 ±16.2	57.5 ±28.7	48.0 ±26.4	49.0 ±18.8	52.1 ±14.1

**Table 4:** Influence of clinical characteristics on health-related quality of life of the study population Values are presented as mean ± standard deviation (analysis performed using t-test, one-way analysis of variance, and Tukey post hoc test) *Comparison of two groups: *HbA1c: <7% vs ≥7%; *hypertension: yes vs no; *dyslipidemia: yes vs no; *deformity: yes vs no; *previous amputation: yes vs no Comparison of more than two groups: BMI: #normal vs overweight, †normal vs obese, ‡overweight vs obese; complications: #one complication vs two complications, †one complication vs three complications, ‡two complications vs ≥three complications HbA1c: glycosylated hemoglobin; BMI: body mass index

Variable	Category	Physical functioning	Role physical health	Role emotional	Energy	Emotional	Social	Pain	General health	Health change
HbA1c	<7%	39.2 ±29.2	27.4 ±13.4	47.6 ±36.6	52.8 ±24.0	71.8 ±26.6	61.9 ±31.4	65.6 ±24.3	57.8 ±18.2	51.9 ±20.9
	≥7%	39.9 ±29.0	26.7 ±18.6	40.0 ±34.5*	50.2 ±23.8	64.0 ±22.2	63.3 ±29.4	59.5 ±29.3	56.5 ±19.1	50.0 ±20.8
Hypertension	Yes	34.9 ±27.7	24.6 ±17.2	43.5 ±35.0	49.4 ±22.1	66.7 ±22.0	60.8 ±29.2	57.7 ±27.9	55.7 ±18.8	49.1 ±20.1
	No	52.7 ±28.5*	32.9 ±17.3	37.8 ±35.1	45.7 ±27.8	64.1 ±27.6	68.7 ±31.2	61.5 ±28.5	60.0 ±18.7	54.1 ±22.5
Dyslipidemia	Yes	36.7 ±29.3	24.6 ±15.1	41.3 ±34.9	50.3 ±23.6	66.4 ±23.4	59.3 ±30.1	57.0 ±28.0	56.4 ±17.3	49.0 ±20.1
	No	54.2 ±22.3*	37.8 ±25.4	45.4 ±36.2	53.5 ±24.7	64.0 ±24.9	80.3 ±21.2*	67.1 ±27.3	58.9 ±25.3	57.6 ±22.6
BMI	Normal	56.7 ±27.9	43.8 ±22.8	51.7 ±33.9	49.0 ±27.6	60.6 ±27.0	56.3 ±33.3	54.4 ±32.4	55.6 ±20.5	53.5 ±26
	Overweight	35.9 ±27.9#	12.1 ±11.8#	24.7 ±19.2#	46.5 ±28.2	59.1 ±27.1	57.6 ±26.6	58.9 ±28.3	53.2 ±19.5	43.5 ±19.6
	Obese	33.2 ±27.0†	27.1 ±17.3	47.4 ±36.8‡	54.6 ±18.1	73.1 ±17.1†‡	69.7 ±29.0	60.9 ±25.8	59.7 ±17.5	53.2 ±17.7
Complications	1 complication	52.0 ±25.7	29.0 ±19.3	35.9 ±22.9	58.2 ±28.1	68.3 ±26.4	68.5 ±25.5	70.8 ±21.6	62.5 ±19.6	55.7 ±19.5
	2 complications	43.6 ±30.8	42.4 ±41.7	51.8 ±36.7	54.6 ±22.8	68.6 ±23.4	65.9 ±29.1	62.2 ±31.9	58.9 ±17.1	56 ±22.9
	≥3 complications	24.3 ±18.9†‡	8.4 ±8.3†‡	37.1 ±24.6	40.2 ±16.2	61.2 ±20.9†‡	54.6 ±33.3	43.9 ±22.5	49.4 ±18.1†‡	39.9 ±15.3†‡
Deformity	Yes	34.8 ±28.1	21.6 ±14.7	40.9 ±24.2	50.7 ±21.9	63.6 ±22.5	58.5 ±30.8	55.3 ±28.3	51.1 ±16.9	74.1 ±20.4
	No	45.5 ±29.1	33.2 ±19.4	43.3 ±26.2	51.0 ±26.0	68.8 ±24.7	68.2 ±28.0	62.7 ±27.4	63.7 ±18.8*	54.6 ±20.6
Previous amputation	Yes	36.4 ±27.7	23.0 ±14.4	41.6 ±24.2	49.3 ±27.4	62.8 ±26.3	57.2 ±32.5	59.5 ±32.0	50.3 ±15.1	47.5 ±22.1
	No	41.1 ±29.5	28.5 ±18.4	42.1 ±25.5	51.5 ±22.2	67.3 ±22.3	65.3 ±28.5	58.4 ±26.4	59.6 ±19.6*	51.7 ±20.2

Linear regression analysis showed no significant differences among the independent factors (Table 5).

**Table 5:** Results of regression analyses with β-coefficient and 95% CI for SF-36 total quality of life CI: confidence interval; HbA1c: glycosylated hemoglobin; BMI: body mass index; SF-36: Short-Form 36-Item Survey

Variable	β	95% CI		t-value	P-value
		Lower	Upper		
(Constant)	56.563	-1.907	115.034	1.931	.058
Gender	-9.123	-22.692	4.446	-1.342	.184
Age	.554	-5.488	6.597	.183	.855
Marital status	3.279	-13.915	20.473	.381	.705
Education	8.344	-.918	17.605	1.799	.077
Treatment type	-1.755	-7.835	4.325	-.576	.566
Occupation	-5.811	-20.136	8.514	-.810	.421
Income	3.543	-9.692	16.777	.534	.595
Smoking	-1.051	-10.175	8.073	-.230	.819
Duration of diabetes	-4.186	-16.898	8.526	-.657	.513
Family history	-2.704	-22.593	17.184	-.272	.787
HbA1c	3.057	-8.369	14.484	.534	.595
Hypertension	1.240	-11.248	13.728	.198	.843
Dyslipidemia	-2.120	-16.525	12.285	-.294	.770
BMI	1.688	-4.560	7.937	.539	.591

## Discussion

Previous studies on DFU and the heavy burden it poses on Saudi Arabia (where its incidence is in the 11.4-29.7% range) have shown that HRQOL is unfavorably affected by it [6,16,17]. Unfortunately, there is a paucity of data on the detrimental effects of foot ulcers on the HRQOL of diabetes patients in Saudi Arabia, which has inspired the current study. Our objective was to determine and evaluate the HRQOL-related factors in patients with DFU associated with T2DM. The results of the present study showed that the patients with DFU revealed lower HRQOL scores relating to all the eight aspects of the SF-36 and also regarding the additional item (perceived change in health). The results also revealed that the HRQOL scores elicited via the SF-36 questionnaire in the domains of physical health and well-being were lower in those having DFU.

Intensive investigations in the past decade on the manner in which males and females with T2DM differ have demonstrated that women with diabetes had worse HRQOL and mental well-being compared to diabetic men [18-20]. The current study also found that females with DFU tended to show poorer HRQOL compared to men, particularly in the subdomains of physical functioning and the role of physical health. In fact, patients with DFU expressed poor consequences of mental and physical health. Forefoot lesions, larger ulcer size, advanced Wagner grade, and higher frequency of unhealed ulcers were more prevalent in females and may have contributed to their poor HRQOL scores [16]. Besides, another recent study highlighted the fact that females may find health services inaccessible, unavailable, or not conditioned to them, in light of specific cultural milieu or gender bias. Women have several restrictions in Saudi Arabia relating to their autonomy, including the restrictions imposed by the male guardianship system. Apart from this, the prevalent constraints of gender segregation and consequent lack of influence play a role in determining the quality and healthcare outcomes for females in Saudi Arabia [21].

In the current study, BMI was identified as a significant risk factor in the subdomains of physical functioning, role limitations due to physical and emotional health, and emotional well-being. Prior studies have indicated that generally, the patients with T2DM are overweight, obese, sedentary, and often hypertensive. One study demonstrated that in men, obesity was negatively linked to HRQOL through DM. However, in women, obesity was directly related to HRQOL and indirectly to HRQOL through DM [22]. Therefore, it has been proposed that patients pay careful attention to their body weight through weight management/reduction programs and raise their physical activity levels to minimize the risk of developing T2DM-related complications [23].

The present study indicated that age is a crucial factor that affects the HRQOL of patients with diabetes. While one study reported that age did not in any way influence the HRQOL of patients with diabetes [24], another study reported contradictory findings, in which patients below 40 years of age showed notably better QOL compared to patients of other age groups [25]. This study identified that age ranked high among the significant risk factors for physical and emotional functioning and the total QOL. Similarly, the HRQOL of patients with diabetes from low socioeconomic backgrounds having a high-school education or less showed a strong negative impact, particularly in the younger age category [26,27]. The findings of this study also indicated that those patients with low economic status and high-school education or less revealed at least one poor HRQOL consequence. It is noteworthy that many studies pointed to a relationship between the longer duration of diabetes and poor HRQOL, for both types of diabetes. However, contradictory results have also been recorded regarding the association between the duration of diabetes and HRQOL [28]. In the current study, diabetes duration was found to be an important risk factor for the subdomain of physical functioning.

Prior studies have demonstrated that smoking has an association with HRQOL scores. However, the present study identified that barring physical functioning, no other subdomains showed any link between smoking and HRQOL scores. Several studies have reported the association between diabetes complications and HRQOL. But in the present study, on comparing with patients having a single complication, remarkably lower QOL was observed for the subdomains of physical functioning, the roles of physical, emotional, and general health, and total QOL. This may be due to patients being unaware of diabetes foot-risk factors and poor foot-care practices [29,30].

The major limitations of this study comprise a relatively small sample size, a limited number of risk factors examined, and limited social and demographic factors examined. Moreover, the study was performed at a single center, and there was no control group with which to compare the study group results. Hence, our results may not be generalizable to the wider population. Further research studies on a larger scale are required to address these limitations.

## Conclusions

Our study showed that patients with DFU in Saudi Arabia generally revealed lower HRQOL. Also, we believe this study delivers valuable evidence that HRQOL is affected most negatively by diabetic foot problems. Therefore, paying more attention to foot care and foot evaluations is crucial in the prevention of foot-related problems associated with DM. Based on the findings in this study, we believe that a greater focus should be placed on foot care for patients with DFU.
